# Expression of plant-produced anti-PD-L1 antibody with anoikis sensitizing activity in human lung cancer cells *via*., suppression on epithelial-mesenchymal transition

**DOI:** 10.1371/journal.pone.0274737

**Published:** 2022-11-11

**Authors:** Thareeya Phetphoung, Ashwini Malla, Kaewta Rattanapisit, Nuttapat Pisuttinusart, Naruechai Damrongyot, Keerati Joyjamras, Pithi Chanvorachote, Tanapati Phakham, Tossapon Wongtangprasert, Richard Strasser, Chatchai Chaotham, Waranyoo Phoolcharoen

**Affiliations:** 1 Graduate Program of Pharmaceutical Sciences and Technology, Faculty of Pharmaceutical Sciences, Chulalongkorn University, Bangkok, Thailand; 2 Center of Excellence in Plant-produced Pharmaceuticals, Chulalongkorn University, Bangkok, Thailand; 3 Department of Pharmacognosy and Pharmaceutical Botany, Faculty of Pharmaceutical Sciences, Chulalongkorn University, Bangkok, Thailand; 4 Baiya Phytopharm Co., Ltd., Bangkok, Thailand; 5 Department of Biochemistry and Microbiology, Faculty of Pharmaceutical Sciences, Chulalongkorn University, Bangkok, Thailand; 6 Pharmacology and Toxicology Unit, Department of Medical Science, Faculty of Science, Rangsit University, Pathum Thani, Thailand; 7 Department of Pharmacology and Physiology, Faculty of Pharmaceutical Sciences, Chulalongkorn University, Bangkok, Thailand; 8 Center of Excellence in Cancer Cell and Molecular Biology, Faculty of Pharmaceutical Sciences, Chulalongkorn University, Bangkok, Thailand; 9 Center of Excellence in Systems Biology, Faculty of Medicine, Chulalongkorn University, Bangkok, Thailand; 10 Excellence Center for Cancer Immunotherapy, Faculty of Medicine, Chulalongkorn University and King Chulalongkorn Memorial Hospital, Bangkok, Thailand; 11 Excellence Chulalongkorn Comprehensive Cancer Center, King Chulalongkorn Memorial Hospital, Bangkok, Thailand; 12 Department of Applied Genetics and Cell Biology, University of Natural Resources and Life Sciences, Vienna, Austria; Hamad Medical Corporation, QATAR

## Abstract

Immune checkpoint antibodies in cancer treatment are receptor-ligand pairs that modulate cancer immunity. PD-1/PD-L1 pathway has emerged as one of the major targets in cancer immunotherapy. Atezolizumab, the first anti-PD-L1 antibody approved for the treatment of metastatic urothelial, non-small cell lung, small cell lung and triple-negative breast cancers, is produced in Chinese Hamster Ovary (CHO) cells with several limitations i.e., high-production costs, low-capacity yields, and contamination risks. Due to the rapid scalability and low production costs, the transient expression in *Nicotiana benthamiana* leaves was investigated by co-infiltration of *Agrobacterium tumefaciens* GV3101 cultures harboring the nucleic acid sequences encoding for Atezolizumab heavy chain and light chain in this study. The transient expression of Atezolizumab in transformed *N*. *benthamiana* accumulated up to 86.76 μg/g fresh leaf weight after 6 days of agroinfiltration (OD 600 nm: 0.4) with 1:1 ratio of heavy chain to light chain. The structural and functional characteristics of plant-produced Atezolizumab was compared with commercially available Tecentriq^®^ from CHO cells with similar binding efficacies to PD-L1 receptor. The direct anti-cancer effect of plant-produced anti-PD-L1 was further performed in human lung metastatic cancer cells H460 cultured under detachment condition, demonstrating the activity of anti-PD-L1-antibody on sensitizing anoikis as well as the suppression on anti-apoptosis proteins (Bcl-2 and Mcl-1) and modulation of epithelial to mesenchymal regulating proteins (E-cadherin, N-cadherin, Snail and Slug). In conclusion, this study manifests plants as an alternative cost-effective platform for the production of functional monoclonal antibodies for use in cancer therapy.

## Introduction

Cancer is a common cause of fatality. Adenoid cystic carcinoma of lung, breast, colorectum and prostate are the most commonly occurring cancers worldwide. Although, chemotherapy is widely used for cancer treatment, there are few limitations such as side effects, use of expensive treatment methods and to overcome therapeutic plateau [1]. Cancer immunotherapy has emerged as a promising technology in the last few decades from the knowledge that tumors are specifically recognized and eradicated by host immune system, leading to the discovery of effective and innocuous treatments. The Food and Drug Administration (FDA) approved the various approaches of cancer immunotherapy that consists of cell therapy, cancer vaccines, immunomodulators, oncolytic virus and immune checkpoint antibodies [2]. Generally, adaptive immunity produces tumor antigen-specific T-cells that can destroy cancerous cells. Immune checkpoints (ICs), e.g., CTLA-4, PD-L1, PD-1 and others, are receptor-ligand pairs that are efficient to modulate cancer immunity [3–5]. However, cancerous cells also use the immune evasion pathway to escape from the host immune system. The PD-1/PD-L1 pathway has been a keen interest for use in cancer immunotherapy. The binding of PD-1 receptor on T-cell to its ligands on cancer cell, lead to deactivation and apoptosis of T-cells [6, 7].

Several immune checkpoint inhibitors that are approved by FDA have shown to have significant clinical benefits in several tumor types [8]. The programmed cell death receptor ligand 1 (PD-L1) also known as B7-H1 or CD274 consists of 290 amino acids, belongs to the group of type I transmembrane protein receptors in B7 family and is naturally present on T-cells, B-cells, monocytes and antigen presenting cells (APC’s) [9]. The PD-1/PD-L1 checkpoint signaling is the key pathway for the tumor cells to evade immune scrutiny [9]. Hence, the use of immune checkpoint blockers has gained critical interest in the recent times from different types of cancer patients with tumor cell or infiltrating immune cell population expressing PD-L1 ligands [10]. Atezolizumab is a highly specific humanized IgG1 antibody, that can block the interaction of PD-L1 ligand expressed on tumor cells with the PD-1 receptors on T-cell population thereby reducing the immunosuppressive action of tumor inducing cells and enhancing the adaptive immune responses in the host against tumors [11]. This FDA approved monoclonal antibody was used as the first-line drug in the treatment of metastatic non-small cell lung cancerous cells (NSCLC’s) with moderate or high PD-L1 expression. The treatment with Atezolizumab has showed significant increase in survival time periods when compared to chemotherapy. Furthermore, it can be used in combination with chemotherapy, cell therapy or other treatments in order to improve the clinical responses [12–14].

Atezolizumab, a humanized monoclonal non-glycosylated antibody, was produced in Chinese Hamster Ovary (CHO) cells [15]. The production of recombinant complex proteins in mammalian cell lines have been proved as the potential expression system with several limitations i.e., high-production costs, low-capacity and contamination risks [16]. Alternatively, plant expression systems have gained major interests in the making of recombinant proteins due to the economical production costs in upstream processes, rapid scalability employing versatile growth conditions, pathogen free with low risks of contamination [3, 17]. *Nicotiana* genus has been widely used establishing a history in the production of heterologous recombinant proteins that include antibodies [18–20], vaccines [21, 22], enzymes [23, 24], growth factors [25–27], diagnostics [28], anti-microbial peptides [29] and other value-added products [30] because of the ease in genome manipulation and fast growth rate [31, 32]. The transient expression by agroinfiltration was adopted in plants to produce various proteins for therapeutic use. *Agrobacterium* mediated gene transfer incorporates the gene of interest in the T-DNA region of the plant cell for protein expression [33].

Atezolizumab, the first line therapy approved globally for metastatic non-small cell lung cancer involves high treatment costs. The current study aimed to use *N*. *benthamiana* as the host system for the transient expression of variable region of Atezolizumab, an anti-human PD-L1 antibody. The plant expressed anti-PD-L1 proteins were characterized for their physicochemical and functional attributes in comparison with the commercial Atezolizumab (Tecentriq^®^). The *in vitro* results obtained showed similar binding affinities to PD-L1 protein, stimulating immune responses. The further studies on non-small cell lung cancer cells with the plant produced anti-PD-L1 was potentially examined for cytotoxicity, cell viability, mode of cell death and expression of various regulatory proteins involving in anoikis, a detachment-induced apoptosis. Hence, this study forms the proof of concept for the use of plant-produced monoclonal antibodies in cancer therapy, especially at metastatic stage.

## Materials and methods

### Construction of recombinant vector

**Fig 1:**
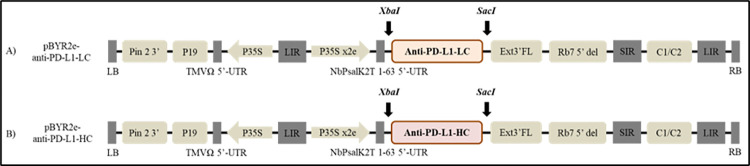
Schematic diagram of T-DNA regions of geminiviral vector (pBYR2e) used in the present study for expression of plant-produced anti-PD-L1 antibody. Pin 2 3’: Potato proteinase inhibitor II terminator, P19: P19 silencing suppressor from Tomato Bushy Stunt Virus (TBSV), TMVΩ 5’-UTR: 5’ untranslated region (UTR) of Tobacco Mosaic Virus Ω, P35S: 35S promoter from Cauliflower Mosaic Virus (CaMV), P35Sx2e: 35s promoter from Cauliflower Mosaic Virus with duplicated enhancer, NbP 5’: 5’ UTR of *Nicotiana* photosystem I reaction center subunit *psa* K, *Xba*I and *Sac*I: restriction enzyme sites for cloning gene of interest, anti-PD-L1-LC: light chain of anti-PD-L1 gene, anti-PD-L1-HC: heavy chain of anti-PD-L1 gene, Ext3’FL: expressed sequence tags- 3’ full length of tobacco extension gene, Rb7: Tobacco Rb7 promoter, C2/C1: Bean Yellow Dwarf Virus (BeYDV) ORFs C1 and C2 which encode the replication initiation protein (Rep) and *Rep*A, LIR: long intergenic region of the BeYDV genome, SIR: short intergenic region of the BeYDV genome, LB and RB: the left and right borders of the *Agrobacterium* T-DNA region.

The amino acid sequences encoding Atezolizumab (Drugbank accession number: DB11595) were codon optimized *in silico* using GeneArt gene synthesis software (Thermo Scientific, MA, USA) for the expression of anti-PD-L1 antibody in plant system. Variable regions of Atezolizumab light chain (LC) and heavy chain (HC) that were added individually to human IgG_1_ kappa chain (Genbank accession number: AAA58989.1) and gamma chain (Genbank accession number: AAA02914.1) were flanked with signal peptide on the N-terminus and a SEKDEL (Ser-Glu-Lys-Asp-Glu-Leu) sequence on C-terminus. Anti-PD-L1 light chain (anti-PD-L1-LC) and anti-PD-L1 heavy chain (anti-PD-L1-HC) that are synthesized were used for cloning into the geminiviral vector pBYR2eK2Md (pBYR2e) by double digestion with *Xba*I and *Sac*I restriction enzymes (BioLabs, MA, USA) separately (Fig 1). The constructed plant expression vectors were transformed into *Escherichia coli* DH10B by heat shock method. Selected colonies were confirmed by colony polymerase chain reaction (PCR) with the primers mentioned in Table 1 and further by sequencing. Confirmed clones were cultured in Luria Bertani (LB) media (HiMedia Laboratories, Mumbai, India) with 50 μg/mL kanamycin (AppliChem, Dermstadt, Germany) overnight at 37°C and the plasmids were isolated and transformed into *Agrobacterium tumefaciens* GV3101 strain by electroporation.

**Table 1:** Primers used in the present study to confirm the selected bacterial clones.

Primer	Sequence
Forward primer: *XbaI*-SP	5’ TCTAGAACAATGGGCTGG
Reverse primer: *SacI*-KD	5’ CGAGCTCTCAAAGCTCATCCTTCTCAGA

### Transient expression of anti-PD-L1 antibody in *Nicotiana benthamiana*

The cultures of *A*. *tumefaciens* expressing light chain and heavy chain of anti-PD-L1 antibody were grown overnight in Luria Bertani (LB) broth with 50 μg/mL of rifampicin (TOKU-E, WA, USA), 50 μg/mL of gentamicin (AppliChem, Dermstadt, Germany) and 50 μg/mL of kanamycin at 28°C. The overnight cultures of *A*. *tumefaciens* harboring *pBYR2e*:*anti-PD-L1 LC* and *pBYR2e*:*anti-PD-L1 HC* were used for agroinfiltration. The *Agrobacterium* cell suspensions containing light chain and heavy chain of anti-PD-L1 were diluted with infiltration buffer (10 mM 2-(N-morpholino)-ethanesulfonic acid (MES) and 10 mM MgSO_4_, pH 5.5) to get the final OD of 0.2, 0.4, 0.6 and 0.8 at 600 nm. The ratio of light chain and heavy chain used for agroinfiltration was 1:1 and each optical density (OD) concentration was co-infiltrated into the leaves of *N*. *benthamiana* plants. The infiltrated leaves were collected on day 2, 4, 6, 8 and 10 post-infiltrations to examine the expression levels of anti-PD-L1. After the analysis of optimum OD and days post infiltration (dpi), the ratio of heavy chain to light chain concentration was further varied to 1:1, 1:2, and 2:1. For optimization of antibody expression, the leaves were infiltrated with a syringe without a needle in three individual plants. Then, the optimized conditions were used in large-scale production by using a vacuum chamber for co-infiltration of anti-PD-L1 heavy and light chains.

### Purification of plantproduced Atezolizumab

The leaves from the optimization experiments were collected and extracted in extraction buffer (5 mM Imidazole, 20 mM Tris-HCl, 50 mM NaCl, pH 7.4), using an electric drill. The samples were centrifuged at 13,000 rpm for 15 min and the supernatant collected was used for quantification of the antibody by ELISA. In large-scale production, the leaves collected were extracted using a blender. The crude extract was centrifuged at 13,000 rpm with the temperature maintained at 4°C for 45 min. The clear supernatant obtained was filtered with a sterile 0.45 μm filter and purified with protein-A affinity chromatography. The column was equilibrated with PBS buffer (pH 7.4) before sample loading. The antibody specifically bound to protein-A beads and then the proteins were washed with PBS buffer. The recombinant antibody was eluted with 0.1 M glycine at pH 2.5 and immediately neutralized with 1.5 M Tris-HCl (pH 8.8). Finally, the purified antibody was 0.22 μm filter sterilized and buffer exchanged with PBS (pH 7.4) in 50 kDa Amicon^®^ ultra-15 centrifugal filter at 4°C, 13,000 rpm for 10 min.

### Confirmation of anti-PD-L1 antibody by SDS-PAGE and western blot

The purified anti-PD-L1 protein was analyzed by sodium dodecyl sulfate-polyacrylamide gel electrophoresis (SDS-PAGE) and western blotting with commercial Tecentriq^®^ as positive control. The samples were independently mixed with reducing buffer (125 mM Tris-HCl pH 6.8, 12% (w/v) SDS, 10% (v/v) glycerol, 0.001% (w/v) bromophenol blue, 22% (v/v) β-mercaptoethanol) and non-reducing buffer (125 mM Tris-HCl pH 6.8, 12% (w/v) SDS, 10% (v/v) glycerol, 0.001% (w/v) bromophenol blue). The samples were separated on 8% SDS-PAGE and then visualized by InstantBlue^TM^ (Expedeon, UK). For western blot, the antibodies were transferred on to nitrocellulose membrane (Bio-Rad, CA). The membrane was blocked with 5% skim milk for 1 h and washed with PBST. The proteins were separately detected using sheep anti-human kappa-HRP and sheep anti-human gamma-HRP conjugated antibodies (The Binding Site, Birmingham, UK). The membranes were washed, developed with electrochemiluminescence (ECL) plus detection reagent (Abcam, UK) and the signal was recorded on medical X-ray green (MXG) film (Carestream, US).

### Size exclusion chromatography

Ultra-high performance liquid chromatography-size exclusion chromatography (UHPLC-SEC) purification system was used to analyze the protein purity and its clusters. In brief, antibody samples (1 mg/mL, 10 μL) were loaded onto 4.6 x 300 mm, 2.5 μM particle size SEC BEH 200 column (Waters, MA, USA). The protein was eluted with PBS buffer (pH 7.4) at a flow rate of 0.3 mL/min. The samples were run for 20 min with column temperature maintained at 25°C. The UV absorbance signals were monitored at 280 nm with Acquity Arc UHPLC system (Waters, MA, USA).

### Quantification of anti-PD-L1 antibody by sandwich ELISA

The plant-produced anti-PD-L1 antibody was quantified by sandwich enzyme-linked immunosorbent assay (ELISA). Briefly, 96-well plates (Greiner Bio-One, Kremsmunster, Austria) were coated with 50 μL of 1:1,000 dilution of goat anti-human IgG and were incubated overnight at 4°C. Then the plates were washed and blocked with 5% skim milk in PBS for 2 h at 37°C. The samples were diluted in a range of 1:1,500 to 1:100,000 for optimization and the quantification of purified antibody. 50 μL of diluted samples were added into each well and incubated for 2 h at 37°C, where human IgG_1_ (Abcam, Cambridge, UK) was used as control to generate the standard curve. A 1:1,000 dilution of sheep anti-human kappa-HRP (The binding site, Birmingham, UK) was added and incubated for 1 h at 37°C after washing with PBST. The plates were developed by adding 50 μL of 3,3’,5,5’-Tetramethylbenzidine (TMB) substrate (Promega, US), followed by 1M H_2_SO_4_ for terminating the reaction. Finally, the plates were measured at an OD of 450 nm by using SpectraMax M5 microplate reader.

### N-glycan analysis

In order to evaluate the authentic glycan structures with quick and sensitive quantification, *N-* glycan analysis of anti-PD-L1 antibody was carried out by liquid chromatography-electrospray ionization-mass spectrometry (LC-ESI-MS) in comparison with non-glycosylated Atezolizumab. Briefly, the heavy chain from purified anti-PD-L1 antibody that was separated on SDS-PAGE, was excised from the gel, S-alkylated and digested with trypsin. The peptides and glycopeptides were trapped on a 30 × 0.32 mm Aquasil C18 pre-column (Thermo Scientific, MA, USA), and then separated on a 100 × 0.18 mm Biobasic C18 analytical column (Thermo Scientific, MA, USA). Positive ions in the range of m/z = 500–1600 were monitored with a Q-TOF Ultima Global mass spectrometer (Waters, MA, USA).

### Human PD-L1 specific binding assay

The specificity of the plant-produced anti-PD-L1 antibody with human PD-L1 was evaluated by ELISA. Briefly, the MaxiSorp high protein-binding capacity 96-well ELISA plate (Thermo Scientific MA, USA) was coated with 100 μL/well of recombinant human PD-L1 His-tag protein (R&D Systems, MN, USA) at a concentration of 0.2 μg/mL, overnight at 4°C. The plate was washed three times and blocked with PBST. Two-fold serial dilutions of Tecentriq^®^, plant-produced anti-PD-L1 antibody, and human IgG_1_ (Novus Biologicals, CO, USA) starting from 2 μg/mL were added to the plate (100 μL/well) and incubated for 1 h at 37°C. Then, HRP-conjugated goat anti-human IgG Fc-γ specific antibody (Jackson Immuno Research, PA, USA), diluted (1:10,000) in PBST (100 μL/well) was added and incubated for 1 h at 37°C. The plate was washed and developed with 100 μL/well of SIGMAFAST™ OPD substrate solution in the dark for 20 min at room temperature. The reaction was stopped by adding 50 μL/well of 1M H_2_SO_4_ and the absorbance was measured at 492 nm using a Cytation™ 5 cell imaging multi-mode reader.

### PD-1/PD-L1 blockade bioassay

The potential neutralization of PD-1/PD-L1 receptor binding with plant-produced anti-PD-L1 antibody was analyzed by cell-based luciferase reporter assay (PD-1/PD-L1 Blockade Bioassays, Promega, WI, US) following the manufacturer’s instructions. Briefly, PD-L1 aAPC/CHO-K1 cells were seeded into flat-clear bottom 96-well assay plate and incubated at 37°C in a 5% CO_2_ incubator for 16 h. Serial dilutions of purified plant-produced anti-PD-L1 antibody and Tecentriq^®^ were added into the plate followed by addition of PD-1 effector cells to each well. The plate was kept in 5% CO_2_ incubator at 37°C for 6 h. The Bio-Glo^TM^ reagent was added to the plate and incubated at room temperature for 5 min after co-culture. The Luminescence signal was measured as relative light units (RLUs) using Cytation™ 5 cell imaging multi-mode reader.

### Cell-based assays

#### Cell culture/chemical reagents

The cell culture reagents that include Roswell Park Memorial Institute (RPMI) 1640 medium, phosphate-buffered saline (PBS) pH 7.4, l-glutamine, fetal bovine serum (FBS) and penicillin/streptomycin solution were procured from Gibco (MA, USA). All other chemical reagents used for cell-based assays, including (4,5-dimethylthiazol-2-yl)-2,5-diphenyltetrazolium bromide (MTT), 2, 3-Bis-(2-Methoxy-4-Nitro-5-Sulfophenyl)-2H-Tetrazolium-5-Carboxanilide (XTT), cisplatin, Hoechst 33342, propidium iodide (PI), dimethysulfoxide (DMSO), crystal violet solution (1% w/v), and formaldehyde solution (37% w/v) were purchased from Sigma-Aldrich Corporation (MO, USA). Annexin V-FITC apoptosis detection kit, SuperSignal^®^ West Pico chemiluminescent substrate and Pierce bicinchoninic acid (BCA) protein assay kits were obtained from Thermo Fisher Scientific (IL, USA). Primary antibodies specific to Mcl-1, Bcl-2, N-cadherin, E-cadherin, Slug, Snail, β-actin and the appropriate horseradish peroxidase (HRP)-linked secondary antibodies were acquired from Cell Signaling Technology, Inc. (MA, USA).

#### Cell culture

Human non-small-cell lung cancer H460 cells (NSCLCs–American Type Culture Collection, VA, USA) were grown in RPMI 1640 medium supplemented with 10% FBS, 2 mM/L l-glutamine, and 100 U/mL penicillin/streptomycin in a humidified 5% CO_2_ incubator at 37°C. When 70–80% of the cell confluency was attained, they were used for further experiments.

#### Cell viability assays

Cell viability of lung cancer cells cultured at attachment condition was measured by MTT assay. A 96-well cell culture plate (Corning, MA, USA) was seeded with H460 cells at a density of 1 × 10^4^ cells/well and incubated for 24 h at 37°C in a 5% CO_2_ incubator. 20 μL of purified anti-PD-L1 antibody was added into the plate at different concentrations (0, 0.1, 0.5 and 1.0 μg/mL) and the cells were further incubated for 24 h. Then, the cells were washed and incubated with 0.4 mg/mL of MTT solution at 37°C for 3 h in dark place. When purple formazan crystals were formed, 100 μL of DMSO was added to dissolve the crystals. The plates were read for OD of formazan solution using a microplate reader (Anthros, NC, USA) at wavelength of 570 nm. The results were expressed as percentage (%) of cell viability by calculating the OD ratio of treated to untreated control cells.

For determining viability under the detached condition, single cell suspension of H460 cells at 1.5 × 10^5^ cells/mL were cultured with RPMI medium with or without purified anti-PD-L1 antibody at non-toxic concentrations (0.1–1.0 μg/mL) in an ultra-low-attachment six-well plate (Corning, MA, USA) for 0–24 h. After indicated timepoint, the cells were assessed for cell viability *via*., the incubation with 20 μM of XTT at 37°C for 3 h kept from light. The OD at 570 nm of formed formazan product was measured using microplate reader and presented as % cell viability as mentioned above.

#### Determination of cell proliferation *via*., crystal violet assay

Crystal violet staining assay was performed to determine the difference in proliferation of NSCLCs upon treatment with purified anti-PD-L1 antibody, at attachment condition. The H460 cells were seeded at a density of 2 × 10^3^ cells/well in a 96-well plate and incubated for 24 h. The purified anti-PD-L1 antibody was added at different concentrations (0, 0.1, 0.5 and 1.0 μg/mL) and the treated cells were further cultured for 24, 48, and 72 h. After each incubation time, the media was removed, and the detached dead cells were removed with two washes of sterile water. The remaining cells were fixed with 1% (w/v) formaldehyde and then incubated with crystal violet solution (0.05% w/v). After 30 min of incubation, they were washed twice with sterile water and air-dried overnight. 200 μL of methanol was added into each well and incubated with continuous shaking for 20 min at room temperature. OD at 570 nm was measured using a microplate reader. The relative cell proliferation was calculated from the ratio of the OD_570_ at each time point divided by the OD of the untreated control at 24 h.

#### Morphological analysis of cell death

Apoptosis and necrosis in H460 cells were characterized by double staining on living cells with Hoechst 33342 (10 μM) and propidium iodide (PI, 5 μg/mL). After 24 h treatment, the control cells and the anti-PD-L1 antibody-treated cells were directly double stained by incubation with Hoechst 33342/PI solution for 15 min at 37°C. The morphology features of apoptosis and necrosis were observed under a fluorescence microscope (Olympus IX51 with DP70). Hoechst 33342, a blue-fluorescence DNA-specific dye permeable to cell membrane, can stain the pycnotic nuclei in apoptotic cells meanwhile PI, a red-fluorescence dye, is permeant only to dead cells indicating necrosis. The ratio of apoptotic cells to total cell number was calculated.

#### Flow cytometry of annexin V-FITC/PI

Human lung cancer H460 cells at 1.5 × 10^5^ cells/mL were cultured in a six-well ultra-low-attachment plate in the presence of various concentrations of anti-PD-L1 antibody (0.1, 0.5 and 1.0 μg/mL), wild type plant extract and commercial atezolizumab for 24 h. The quantification of apoptotic cells under detachment condition or anoikis was further confirmed by flow cytometry using Annexin V-FITC (fluorescein isothiocyanate)/PI co-staining assay kit. Briefly, at the end of 24 h incubation, the cells were harvested and resuspended in PBS buffer, pH 7.4. Annexin V-FITC/PI staining was performed by following the manufacturer’s instructions. The cell suspensions were centrifuged and resuspended in 100 μL of 1X binding buffer. 10 μL of annexin V-FITC (1 μg/mL) and 5 μL of PI (2.5 μg/mL) were used for staining the cells for 15 min in the dark. Finally, 400 μL of the 1X binding buffer was added and the cells were kept on ice for analysis. Analysis was performed using Guava easyCyte 5 benchtop flow-cytometer. For detecting green fluorescence of Annexin V-FITC (emission wavelength at 518 nm), the violet laser (405 nm) was selected for the excitation wavelength. Meanwhile, the green laser at 532 nm was chosen as the excitation wavelength to evaluate red fluorescence of PI (emission wavelength at 575 nm) *via*., Yellow-B channel. The cells ranged between 2,000–26,000 HL in of both side and forward scatters were gated and counted for 10,000 events for further analysis of mode of cell death via the guavaSoft version 2.7 software (Merck, Darmstadt, Germany).

#### Western blot

The H460 cells at a density of 1.5 × 10^5^ cells/mL in RPMI culture medium were seeded into a six-well ultra-low-attachment plate and treated with different non-toxic concentrations of anti-PD-L1 antibody for 12 h. The cells were collected by centrifugation at 5,000 rpm for 5 min at 4°C and further incubated in radioimmunoprecipitation assay (RIPA) buffer along with protease inhibitor at 4°C for 30 min for efficient lysis of cells and solubilization of proteins. The cell suspensions were centrifuged at 4°C, 10,000 rpm for 7 min and supernatants collected were used for protein quantification by BCA protein assay kit. The equal concentration of proteins (30 μg) was used for SDS-PAGE in reducing condition and then transferred to nitrocellulose membranes (Bio-Rad Laboratories, CA, USA). The membranes were blocked with 5% skim milk in TBST (25 mmol/L Tris- HCl, pH 7.4, 125 mmol/L NaCl, 0.1% Tween 20) for 1 h at ambient temperature. Then, the membranes were probed with primary antibodies of Mcl-1, Bcl-2, N-cadherin, E-cadherin, Slug, Snail and β-actin at 4°C overnight. The membranes were washed with TBST and then incubated with HRP-conjugated secondary antibodies (Cell Signaling) for 1 h at room temperature. The chemiluminescence signals were quantified by Analyst/PC densitometry software (Bio-Rad Laboratories CA, USA). β-actin was used as an internal control.

### Statistical analysis

Results were presented as mean ± standard deviation (SD) from the three independent experiments. The statistical analysis was performed by one-way analysis of variance (ANOVA) and Tukey’s HSD post-hoc test using SPSS version 22 (IBM Corp., NY, USA) to determine the significant differences between experiments and was considered as statistically significant at *p* value < 0.05. Additionally, the validity of data was evidenced with the non-significance values (*p* > 0.05) of normality of residuals and homogeneity of variance prior performing ANOVA analysis.

## Results

### Optimization conditions for anti-PD-L1 expression

**Fig 2:**
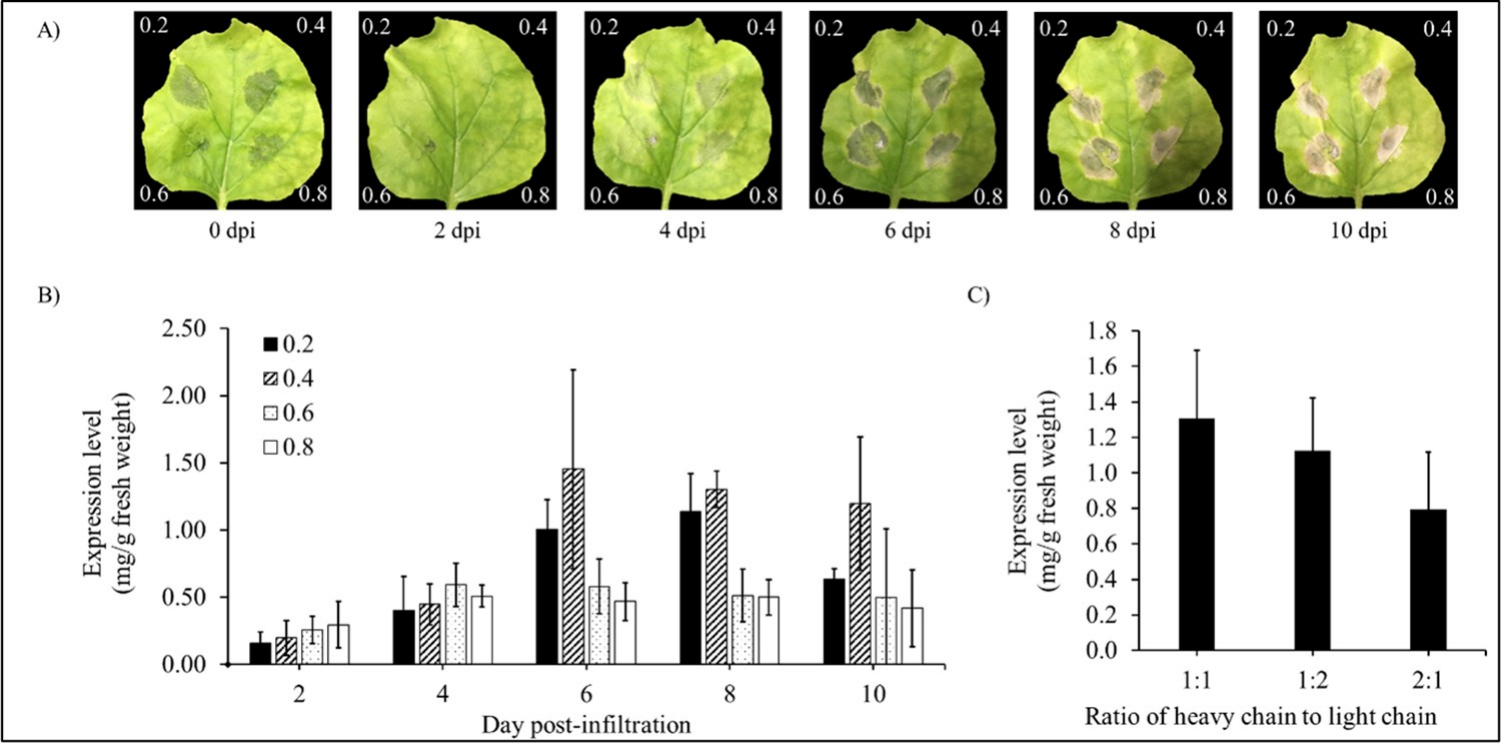
Optimization conditions of optical density (OD) of *Agrobacterium tumefaciens*, days post-infiltration (dpi) and heavy chain to light chain ratio. (A) Plant-expressed anti-PD-L1 antibody at different ODs of *A*. *tumefaciens* on Day 0, 2, 4, 6, 8 and 10 post-infiltrations. The expression levels of anti-PD-L1 antibody were quantified by sandwich ELISA. Data are represented as mean ± SD. (B) Optimization of optical density and days post-infiltration. (C) Optimization of heavy chain to light chain ratio.

To produce anti-PD-L1 antibody in *N*. *benthamiana*, variable regions of Atezolizumab gene were constructed with human IgG_1_ flanked with signal peptide at N-terminal and SEKDEL sequence at C-terminal. The genes of light chain and heavy chain were synthesized and codon-optimized for *N*. *benthamiana* before being cloned individually into the expression vector pBYR2e. After the confirmation of clones by PCR and sequencing, the recombinant plant expression vector pBYR2e harboring the light and heavy chains of Atezolizumab were further transformed into *A*. *tumefaciens* GV3101. The bacterial clones of anti-PD-L1 light chain and anti-PD-L1 heavy chain were cultured individually for co-infiltration into *N*. *benthamiana* leaves. The different parameters such as bacterial culture OD, days post-infiltration (dpi) and heavy chain to light chain ratio was investigated. The infiltrated leaves showed cell necrosis on 4 dpi but, significant necrosis was observed on 6 dpi as shown in Fig 2A. When the leaves were co-infiltrated in the ratio of 1:1, light chain to heavy chain and culture OD of 0.4 at 600 nm, optimum expression level was obtained on day 6 post-infiltration (Fig 2B and 2C).

### Extraction and purification of anti-PD-L1 from *N*. *benthamiana*

**Fig 3:**
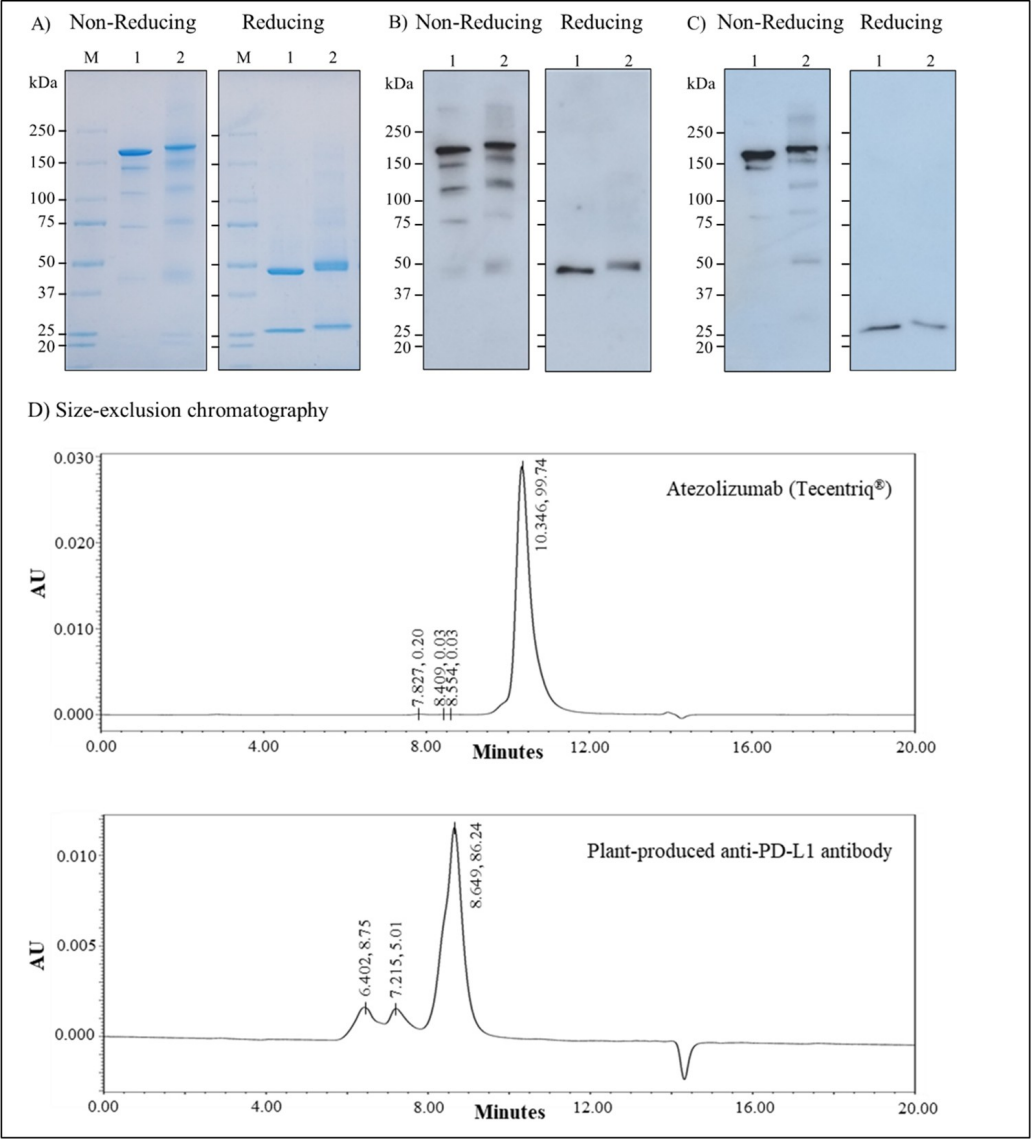
SDS-PAGE and western blotting of purified anti-PD-L1 antibody under non-reducing and reducing conditions. (A) SDS-PAGE analysis by Coomassie staining (B) Western blot analysis with sheep anti-human gamma-HRP (C) Western blot analysis with sheep anti-human kappa-HRP. kDa: Kilodalton, M: Protein marker, Lane 1: Atezolizumab (Tecentriq^®^) (Positive control), Lane 2: anti-PD-L1 antibody (D) Chromatogram of Tecentriq^®^ and plant-produced Atezolizumab by size exclusion chromatography.

In the large-scale production of plant-produced anti-PD-L1, the *N*. *benthamiana* plants were co-infiltrated with *Agrobacterium* cultures (OD at 600 nm = 0.4) harboring the heavy chain and light chain in the ratio of 1:1 respectively. The infiltrated leaves were collected after 6 dpi, extracted and purified with protein-A affinity chromatography. The purified antibody was buffer exchanged and then analyzed by SDS-PAGE, western blotting and sandwich ELISA. The anti-PD-L1 protein bands were observed on non-reducing SDS-PAGE at approximately 150 kDa and the heavy chain was observed at ~ 50 kDa and the light chain at ~ 25 kDa under reducing conditions (Fig 3A). Anti-PD-L1 heavy chain and light chain were specifically detected with sheep anti-human gamma-HRP and sheep anti-human kappa-HRP (Fig 3B and 3C). Additionally, size exclusion chromatography was performed to evaluate the protein purity and protein aggregates. Fig 3D demonstrates that the plant produced Atezolizumab assembled as full IgG molecule (major peak) in similar with Tecentriq^®^. The smaller peaks observed relate to the antibody aggregates. The amount of anti-PD-L1 expressed in large-scale production is 2.04 mg/mL or 86.76 μg/g fresh weight by sandwich ELISA quantification.

### N-linked glycosylation pattern of anti-PD-L1 antibody

**Fig 4:**
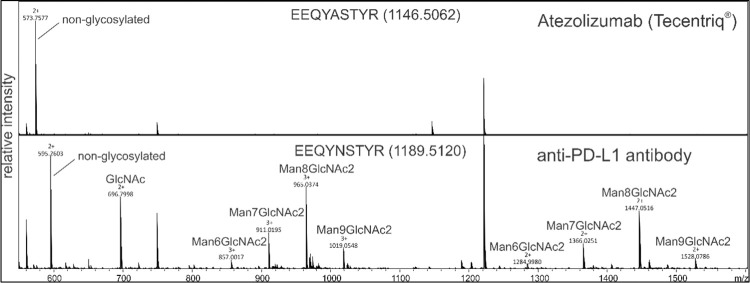
Liquid chromatography-electrospray ionization-mass spectrometry (LC-ESI-MS) of Tecentriq^®^ and plant-produced PD-L1 antibody after trypsin digestion. The N-glycosylation profile of the plant-produced glycopeptide EEQYNSTYR is depicted with other major peaks.

To evaluate the N-glycan profile of plant-produced Atezolizumab, LC-ESI-MS was used. The N-glycosylation pattern of the plant-produced anti-PD-L1 antibody was compared to non-glycosylated Atezolizumab (Tecentriq^®^) (Fig 4), revealing that the anti-PD-L1 antibody produced in *N*. *benthamiana* displayed oligo-mannose glycan residues that are usually required for endoplasmic reticulum enunciated proteins without showing any effect on the binding property of this antibody with its target.

### Functional assays of plant-produced anti-PD-L1 mAb

**Fig 5:**
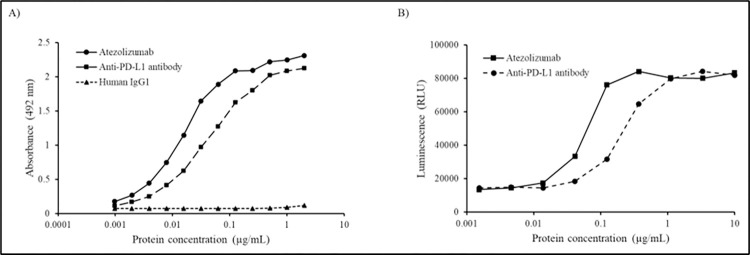
Binding and inhibition assays of anti-PD-L1 antibody. (A) Specific binding to human PD-L1 by ELISA. The Tecentriq^®^ and human IgG_1_ was used as positive and negative control respectively. Plant-produced anti-PD-1 antibody was also used to confirm the non-specificity to PD-L1. (B) PD-1/PD-L1 blockade bioassay for determining the inhibition activity of anti-PD-L1 antibody. Data are represented as mean ± SD of technical triplicates.

The purified plant-produced anti-PD-L1 antibody binding to human PD-L1 protein *in vitro* was determined by ELISA as shown in Fig 5A. The commercial mammalian cell produced and plant-produced anti-PD-L1 mAbs showed similar binding to human PD-L1 protein, while the human IgG1 antibody used as negative control did not show any binding. The luciferase reporter system was used to assess the inhibitory activity of plant-produced anti-PD-L1, where serial dilutions of this antibody were added to PD-L1 aAPC/CHO-K1 cells and the plate was incubated for 6 h. The activation of luciferase expression under the control of nuclear factor of activated T-cell promoter indicates the blocking of PD-L1/PD-1 interaction in the presence of anti-PD-L1 mAb. The results indicated the inhibition of PD-L1 protein interaction with its receptor in both plant-produced and commercial Atezolizumab with EC_50_ values of 0.0565 and 0.2195 μg/mL respectively (Fig 5B) confirming their functionality activity of anti-PD-L1 antibody. Data are represented as mean ± SD of technical triplicates.

### Cytotoxicity of anti-PD-L1 in human non-small cell lung cancer

**Fig 6:**
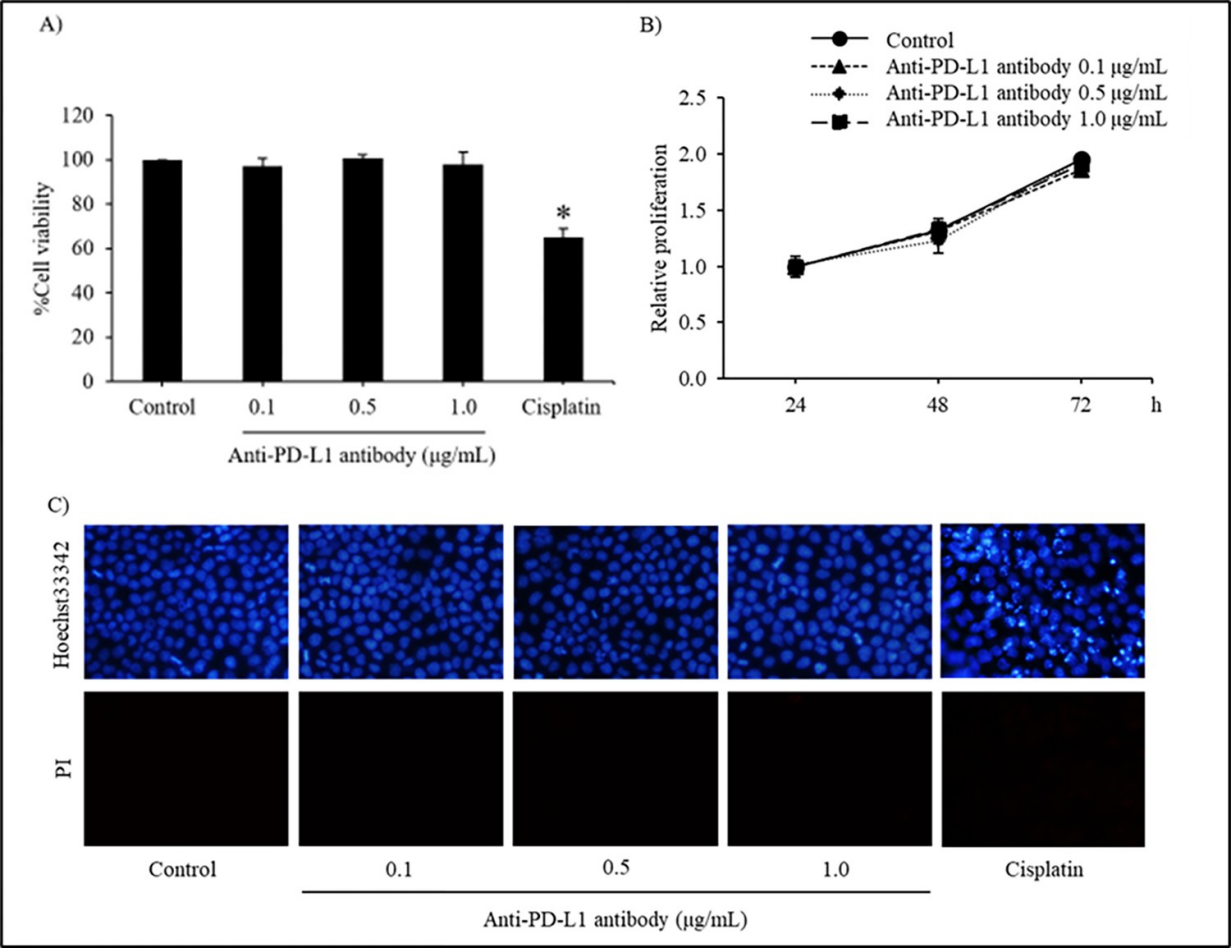
Cytotoxicity of anti-PD-L1 in human non-small-cell lung cancer H460 cells. (A) MTT viability assay tested with 0.1, 0.5 and 1.0 μg/mL of plant-produced anti-PD-L1 (B) Crystal violet staining assay for examining the non-toxic concentrations of anti-PD-L1 on cell proliferation rate (C) Apoptosis and necrosis, which were respectively visualized with blue fluorescence of Hoechst 33342 and red fluorescence of propidium iodide (PI), were barely detected in H460 cells treated with anti-PD-L1 antibody (0–1.0 μg/mL) for 24 h. Cisplatin, an anticancer drug, at 25 μM was used as a positive control for cell viability and mode of cell death assays. Values are means of the independent triplicate experiments ± SD; *p < 0.05 compared with non-treated control cells at same time points.

Because of high rate of incidence and cancer-related death worldwide [34], cytotoxic activity of plant-produced anti-PD-L1 mAb was initially examined on human non-small cell lung cancer cells. To evaluate anoikis sensitizing effect, the non-toxic concentrations of plant-produced anti-PD-L1 were preliminary clarified for further used in next studies. Human lung cancer H460 cells seeded on a 96-well plate were treated with plant-produced anti-PD-L1 at a concentration of 0.1, 0.5 and 1.0 μg/mL for 24 h, with non-treated H460 cells as negative control. After 24 h of incubation period, MTT viability assay showed that plant-produced anti-PD-L1 did not exhibit toxic effect in any of the concentrations tested (Fig 6A) meanwhile treatment with 25 μM cisplatin, a recommended anticancer drug as positive control, significantly reduced viability in lung cancer H460 cells. The non-toxic concentrations were further used to assess the cell proliferation rate as illustrated in Fig 6B. The H460 cells were treated with anti-PD-L1 for 24, 48 and 72 h and were further stained with crystal violet solution. All anti-PD-L1 protein-treated cells exhibited no alteration of proliferation at 24, 48 and 72 h in comparison with the untreated group of cells. Additionally, mode of cell death assessment *via*., Hoechst 33342 and PI co-staining in H460 cells incubated with plant-produced anti-PD-L1 (0–1.0 μg/mL). Though anti-PD-L1-treated cells exhibited neither blue fluorescence which strongly indicates apoptotic cell death nor red fluorescence PI-stained necrosis, culture with cisplatin (25 μM) significantly induced apoptosis in H460 cells after 24 h of incubation time (Fig 6C). Therefore, the non-toxic concentrations ranging between 0.1–1.0 μg/mL of plant-produced anti-PD-L1 were chosen for the further investigation of anoikis sensitizing effect.

### Plant-produced anti-PD-L1 sensitizes anoikis in human NSCLC

**Fig 7:**
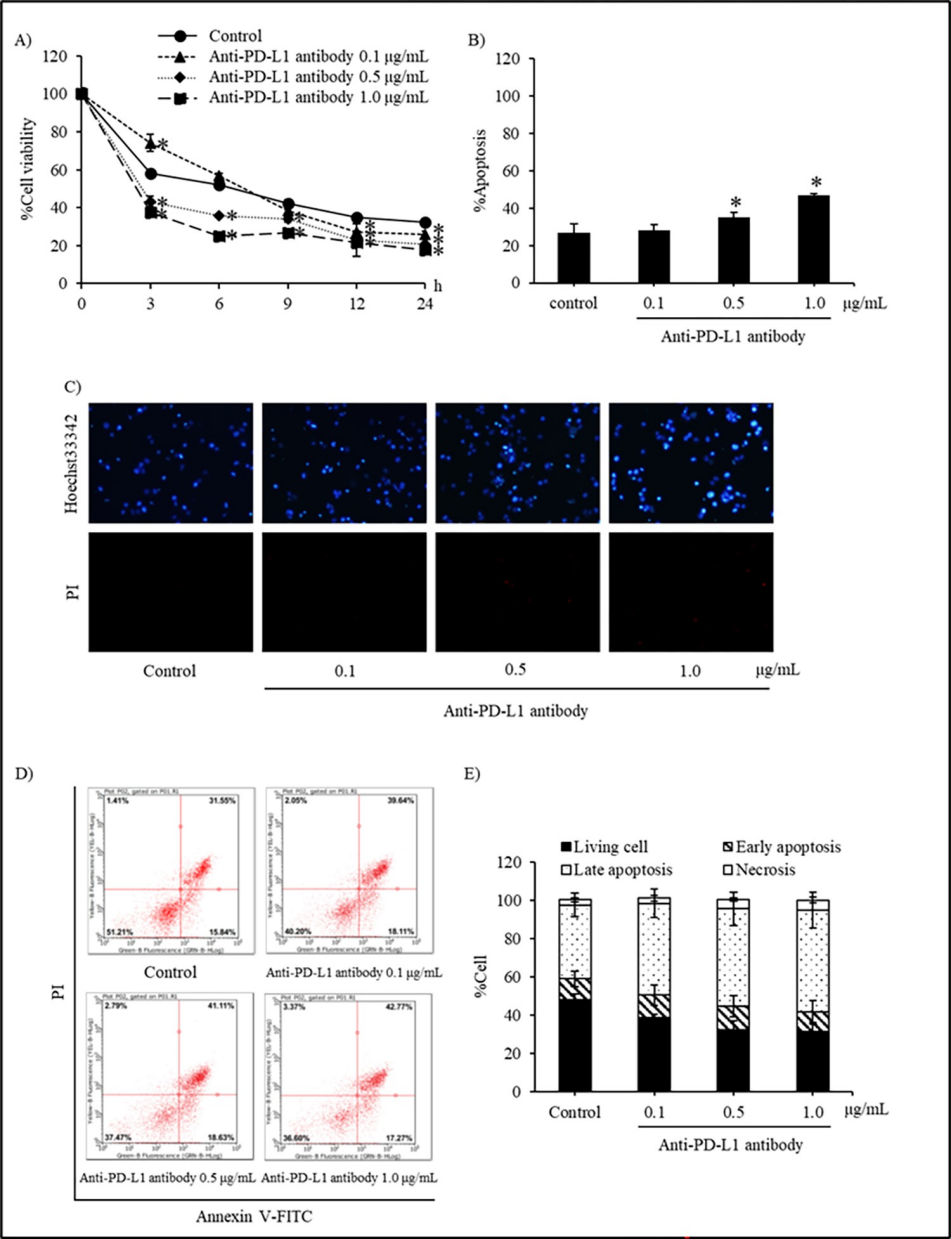
Plant-produced anti-PD-L1 antibody sensitizing detachment-induced cell death in human non-small cell lung cancer H460 cells was evidenced with (A) the reduced % cell viability assessed by XTT assay (B) the accumulated % apoptosis at 24 h of incubation period (C) the induction of apoptosis staining with bright blue fluorescence of Hoechst 33342 after treatment with anti-PD-L1 at 0.1, 0.5 and 1.0 μg/mL for 24 h (D) The histograms from flow cytometry of annexin V-FITC/propidium iodide (PI) also illustrate the living cells (Annexin V-FITC negative/PI negative) presenting anoikis resistant capability in lung cancer H460 cells (E) Interestingly, the percentage of early (Annexin V-FITC positive/PI negative) and late (Annexin V-FITC positive/PI positive) apoptosis obviously increased in the cells incubated with plant-produced anti-PD-L1 antibody (0.1–1.0 μg/mL) for 24 h under the detached condition. Values are means of independent triplicate experiments ± SD; *p < 0.05 compared with non-treated control cells.

Although the treatment with plant-produced anti-PD-L1 antibody (0.1–1.0 μg/mL) did not cause significant cytotoxicity in human non-small cell lung cancer cells cultured under attachment condition (Fig 6), the sensitizing effect on detachment-induced cells that is a crucial step in metastatic process was further evaluated in anti-PD-L1-treated H460 cells. To investigate the anoikis sensitizing activity of plant-produced anti-PD-L1 antibody in detachment condition, H460 cells were treated with non-toxic concentrations (0.1, 0.5 and 1.0 μg/mL) of anti-PD-L1 antibody for determining the cell viability using XTT assay from 0–24 h (Fig 7A). The non-treated H460 cells showed 40% decrease in cell viability in the first 3 h of culturing under the detachment and thereafter attaining a stable viability at 30–40% within the 24 h monitoring period, indicating their capability to resist the cell death due to detachment. The cell viability significantly decreased after 3 h in the treatment groups of 0.5 and 1.0 μg/mL, whereas the 0.1 μg/mL treatment showed a reduction at 12 h when compared with the control cells. Interestingly, treatment for 9–24 h with plant-produced anti-PD-L1 antibody (1.0 μg/mL) and commercial Atezolizumab (1.0 μg/mL) significantly reduced viability in H460 cells when compared to non-treated control cells, while the incubation with wild type plant extract (1.0 μg/mL) showed equal cell reduction as that of control cells during 6–24 h of the incubation time (S1 Fig).

The modes of cell death were analyzed by co-staining with Hoechst 33342 and PI to confirm the anoikis sensitizing activity of plant-produced anti-PD-L1 antibody. After culture for 24 h under detachment condition, lung cancer H460 cells showed the apoptosis without any necrotic cell death as depicted in Fig 7C. Intriguingly, the presence of bright blue fluorescence of Hoechst 33342 indicating apoptosis with condensed DNA and/or fragmented nuclei was obviously increased in H460 cells cultured with anti-PD-L1 antibody. The apoptotic cell death rate was calculated using the ratio of number of apoptotic cells to the total number of cells. Fig 7B displays the augmented apoptotic cell death in concentration dependent manner when the cells were treated with 0.5 and 1.0 μg/mL for 24 h, under detachment condition.

Flow cytometry was further performed to confirm the anoikis in the detached H460 cells. The H460 cells were treated with different concentrations (0, 0.1, 0.5 and 1.0 μg/mL) of anti-PD-L1 antibody for 24 h and then the cells were stained with annexin V-FITC and PI. The cells were tracked for cell death with labeled conditions, *i*.*e*., annexin V-FITC for tracking early apoptosis, annexin V-FITC conjugated with PI for indicating late apoptosis and PI alone for cell necrosis. Correspondence with the determination of cell viability, histograms from flow cytometry present not only the decreased amount of living cells (Annexin V-FITC negative/PI negative) but also the increase of apoptosis at both early (Annexin V-FITC positive/PI negative) and late (Annexin V-FITC positive/PI positive) stage in H460 cells treated with anti-PD-L1 antibody (0.5–1.0 μg/mL) compared with the untreated control cells (Fig 7D). Fig 7E also indicate the dose-dependent augmentation of % apoptosis in anti-PD-L1-treated H460 under the detached condition. It was worth noted that the minor alteration of necrotic cells (Annexin V-FITC negative/PI positive) was illustrated in anti-PD-L1-treated cells. Wild type plant extract and commercial Atezolizumab were also evaluated and the data is shown in S1 Fig.

### Plant-produced anti-PD-L1 antibody modulates EMT regulating proteins

**Fig 8:**
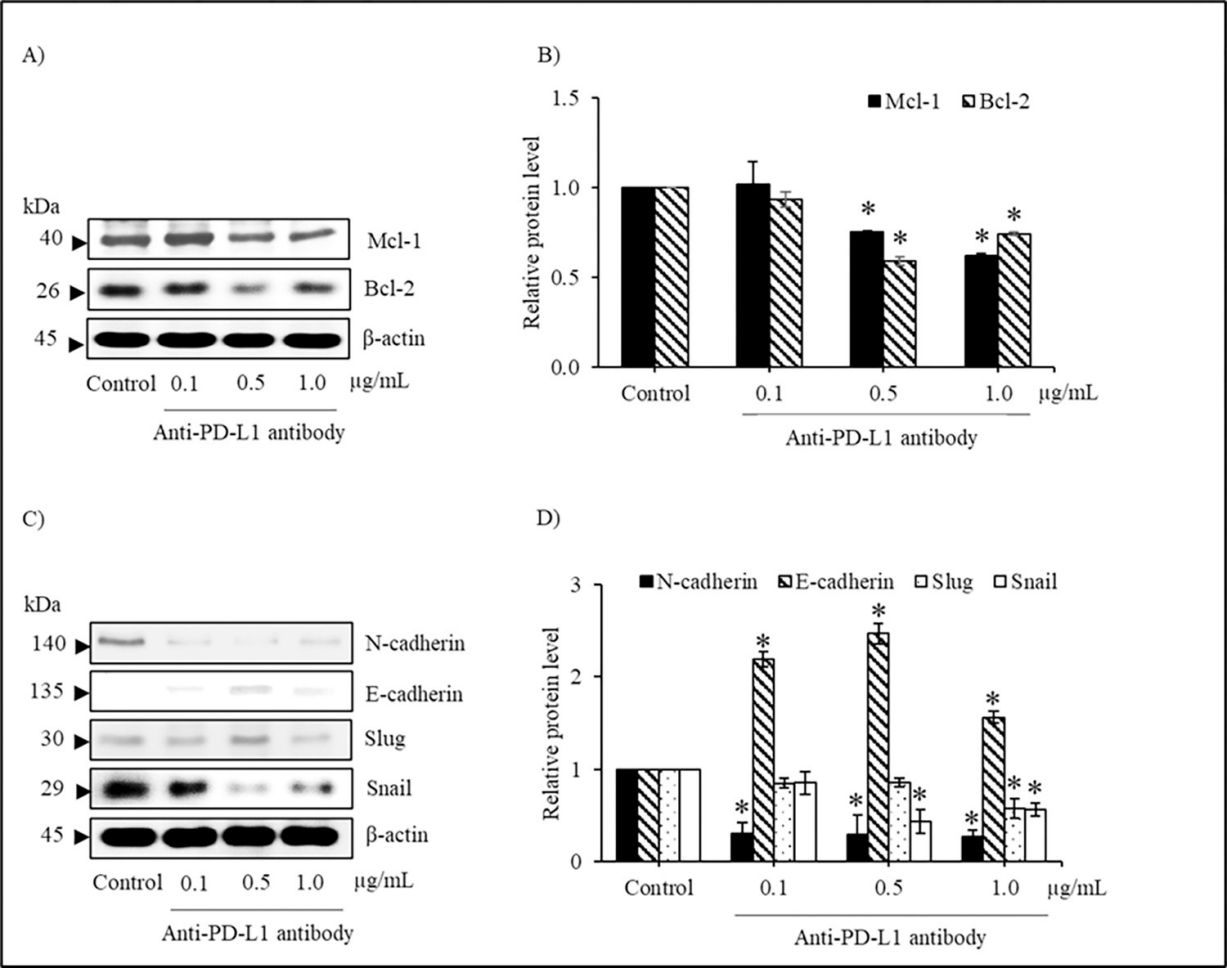
Plant-produced anti-PD-L1 antibody modulates anoikis regulating proteins. (A) The alteration of apoptosis mediating proteins were demonstrated in human lung cancer H460 cells treated with anti-PD-L1 antibody for 12 h under detachment condition via western blot analysis (B) Culture with 0.5–1.0 μg/mL significantly decreased levels of anti-apoptosis, Mcl-1 and Bcl-2, in H460 cells compared with the untreated cells (C) Not only over-expression of E-cadherin but also down-regulation of N-cadherin was illustrated in plant-produced anti-PD-L1-treated H460 cells (D) The signaling proteins mediating epithelial-mesenchymal transition (EMT), including Slug and Snail were diminished in lung cancer H460 cells after incubation with 0.1–1.0 μg/mL anti-PD-L1 for 12 h. Non-treated cells were used as control group and β-actin was used as loading control. Values are means of the independent triplicate experiments ± SD; *p < 0.05 compared with non-treated control cells.

Western blot analysis was used to elucidate the alteration of regulating protein related to anoikis. Lung cancer H460 cells were cultured under detachment condition with or without plant-produced anti-PD-L1 antibody (0.1–1.0 μg/mL) for 12 h. Fig 8A shows the reduction of anti-apoptotic proteins, Mcl-1 (Myeloid cell leukemia-1) and Bcl-2 (B-cell lymphoma 2), in anti-PD-L1-treated H460 cells. It was noted that the significant decrease in the levels of Mcl-1 and Bcl-2 was shown in H460 cells treated with 0.5 and 1.0 μg/mL of anti-PD-L1 in comparison to control cells. Because the transition from epithelial to mesenchymal cells mediates anoikis resistance in lung cancer cells [35], the alteration of proteins related to epithelial-mesenchymal transition (EMT) were also determined in H460 cells incubated with plant-produced anti-PD-L1 antibody. Western blotting obviously shows the increased level of E-cadherin, an epithelial protein maker, in detached H460 cells cultured with 0.1–1.0 μg/mL anti-PD-L1 antibody for 12 h (Fig 8C). The significant diminution of transcription factor proteins mediating mesenchymal features, including Slug and Snail was demonstrated after treatment of anti-PD-L1 antibody at 1.0 and 0.5 μg/mL compared with untreated control cells (Fig 8D). Notably, the incubation with plant-produced anti-PD-L1 at low concentration (0.1 μg/mL) also dramatically down-regulated a protein marker of mesenchymal transition, N-cadherin, in lung cancer H460 cells under detachment condition.

## Discussion

Immune checkpoint blockers (ICBs), as monoclonal antibodies have gained momentum in cancer immunotherapy due to their interference in the binding of immune checkpoint ligands with their receptors, thereby resulting in T-cell immune response activation to eradicate cancer cells [2]. PD-1/PD-L1 checkpoint is associated with autoimmune response of T-cells and interestingly, PD-L1 ligands are up-regulated in many tumor types, including non-small-cell lung cancer at metastatic stage to evade the cytotoxic T-cell population, by imitating their immune responses [7, 36]. Atezolizumab is an IgG_1_ mAb, that specifically binds to PD-L1 ligands on malignant cells to eliminate the cancerous cells [37]. Atezolizumab, produced in mammalian cells was approved as first-line mode of therapy for the commonly occurring metastatic non-small cell lung cancer [14] involving high investment costs, verbose optimizations, sterility risks and stringent manufacturing environments [38].

In the present study, we produced anti-PD-L1 in *N*. *benthamiana* by cloning the Atezolizumab and human IgG1 sequences in geminiviral plant expression vector. Plant expression systems are being routinely used in the recent years for the production of antibodies due to ease in transformation techniques, cost-effective manufacturing and rapid scalability [18]. The plant-produced anti-PD-L1 antibody was investigated in this study for its physicochemical and functional properties and demonstrated its potentiality on metastatic non-small cell lung cancer cells through anoikis induction. The production and expression of immune checkpoint inhibitors in *N*. *benthamiana* proved their efficacy in immunotherapeutics as reported from previous studies [3, 4]. Based on our results, we found that the optimum conditions for producing the plant-produced anti-PD-L1 are bacterial density at 0.4, 6 dpi, and 1:1 of heavy chain to light chain ratio which were used for large-scale production. The presence of high bacterial concentration leads to plant necrosis [39, 40]. With these optimized conditions, the expression level of anti-PD-L1 mAb was found to be 2.04 mg/mL or 86.76 μg/g fresh weight.

The plant-produced anti-PD-L1 was characterized for the chemical entities by elucidating N-glycosylation profile, which depicted the presence of high number of mannose oligosaccharides (Fig 4) attributing to the target signaling in endoplasmic reticulum due to the presence of SEKDEL sequence. The structure of Man_5-9_GlcNAc_2_ is normally seen in N-linked glycans in plants and *N*. *benthamiana* [41, 42] and SEKDEL tagged antibodies have shown high-mannose N-glycans that are non-immunogenic [43]. Although previous reports show that plant-produced antibodies with mannosidic N-glycans have increased rate of antibody clearance from circulation [44] and no allergic reactions or hypersensitivity responses [45, 46], glycan engineered plants are an alternative to overcome these bottlenecks for use in potential therapeutic applications. The functional characteristics of plant-produced Atezolizumab were further assessed for binding activity and binding kinetics by ELISA and PD-1/PD-L1 blockade assay respectively (Fig 5), confirming that this mAb effectively binds to human PD-L1 protein in similarity with Tecentriq^®^ and other earlier findings [3, 4]. The protein aggregates that were observed from the SEC of plant-produced anti-PD-L1 antibody might have interfered showing slightly lower binding with human PD-L1.

Generally, solid tumors form the primary cause of cancer-related transience by distinct steps of biological process known as metastasis, wherein the cells lose their adhesion from the extracellular matrix, evade apoptotic cell death, resist anoikis and are displaced from the primary sites through the lymphatic or circulatory systems for their survival [47, 48]. Resistance to detachment-induced cell death has been considered as a critical step in metastatic process and the sensitizing anoikis is effectively restrained metastatic lung cancer cells [49]. Many studies have shown various molecular pathways of anoikis and its protection in physiological conditions when PD-L1 ligand binding to its receptor is hindered by anti-PD-L1 mAb’s, illustrating the PD-L1 role and function in intracellular signal transduction pathways [50–52]. The expression PD-L1 on cell membrane was reported to correlate with the chemotherapeutic resistance in cancer cells through modulating MAPK/ERK survival pathway. Conversely, treatment with anti-PD-L1 antibody effectively restored the susceptibility to cisplatin, a recommended chemotherapeutic drug, in breast cancer cells [53]. Although anoikis sensitization is not a primary mode of action of Atezolizumab, the capability of EMT suppression of Atezolizumab and the overexpression of PD-L1 on circulating tumor cells [54] strongly suggest the anoikis-sensitizing effect of anti-PD-L1 antibody. The present study focused on cytotoxicity studies, anoikis sensitization and regulatory proteins mediating anoikis.

Cell-based experiments were performed in human non-small cell lung cancer H460 cells, for evaluating the cytotoxicity of plant-produced anti-PD-L1 antibody at both attachment and detachment culture condition. The H460 cells possessing highly metastatic features have been proved as the candidate lung cancer cells for determining the capability of anoikis resistance [55–57] which corresponded with the results in this present study indicating the remaining viable cells approximately 40–50% after culture under detachment condition for 24 h (Fig 7A, 7D and 7E). Despite of no alteration on viability and proliferation of lung cancer cells at attachment culturing (Fig 6), the plant-produced anti-PD-L1 at 0.1, 0.5 and 1.0 μg/mL significantly induced anoikis in H460 cells under detached condition (Fig 7). It was worth noted that plant-produced anti-PD-L1 antibody at 0.5–1.0 μg/mL significantly diminished viable cells under detachment condition early at 3 h (Fig 7A) and induced apoptotic cell death after 24 h (Fig 7B–7E) in comparison with wild type plant extract (S1 Fig), signifying the capability of anoikis sensitization and preventing the formation of metastatic malignant tumors. An *in vivo* study demonstrated that a half-life of PD-L1 antibody approximately at 46.7 h [58]. This information suggests the potential activity of PD-L1 antibody on anoikis sensitization during 12-24-h treatment presented in this study.

Anoikis is modulated by apoptotic proteins of the Bcl-2 family playing a vital role. The anti-apoptotic proteins, Bcl-2 and Mcl-1 are known to thwart the activation of the effector/pro-apoptotic proteins Bak/Bax oligomerization on the outer membrane of mitochondria and sequester the BH3-only proteins, resulting in apoptosis inhibition [50, 59]. The upregulation of Bcl-2 and Mcl-1 shown to involve with anoikis resistance [60–62]. Consequently, both these anti-apoptotic proteins were down-regulated with increasing concentration of anti-PD-L1 mAb (Fig 8A and 8B), which was correlated with augmentation of apoptosis in anti-PD-L1-treated H460 cells, illustrating its potentiality to sensitize anoikis in highly metastatic lung cancer cells.

Epithelial-mesenchymal transition is a process where cancerous epithelial cells undergo many biochemical changes to attain the phenotype of mesenchymal cells, thereby making them resistant to apoptosis, with increased migratory extents and pervasiveness [49, 63]. EMT is characterized by the loss of expression of various epithelial proteins such as E-cadherin along with enhanced expression levels of mesenchymal protein marker like N-cadherin. EMT is also a crucial event for anoikis resistance wherein, down-regulation of E-cadherin and up-regulation of N-cadherin called as E/N cadherin switch promotes metastasis [64–66]. Snail and Slug are zinc-finger transcriptional factors, that are aberrantly expressed on cancer cells inducing EMT and anoikis resistance by the inhibition of E-cadherin transcription [50, 67, 68]. The current study showed up-regulation of E-cadherin and decreased expressions of N-cadherin, Slug and Snail proteins when treated with anti-PD-L1 mAb produced in *N*. *benthamiana*, indicating its functional capacity in impairing EMT and anoikis resistance.

Though the diminution of EMT transcription factors, Snail and Slug, as well as anti-apoptotic Bcl-2 and Mcl-1 proteins was not obviously observed in detached lung cancer cells cultured with anti-PD-L1 at low concentration (Fig 8A and 8C), the dramatic up-regulation of N-cadherin expression level (Fig 8D) and % cell viability (Fig 7A) was demonstrated in the cells incubated with 0.1 μg/mL anti-PD-L1 for 12 h. It has been reported that down-regulated expression of E-cadherin is essential for induction of anoikis [69, 70]. Indeed, the relation between EMT, metastatic stage and PD-L1 expression level has been revealed in various cancers [71–73]. It was found that lung cancer patients with negative expression of PD-L1 and EMT markers owned high survival rate [74, 75]. It has been reported that PD-L1 mediates EMT through inhibition and degradation of Snail. Moreover, treatment with PD-L1 inhibitor sufficiently suppressed the migratory activity through modulating EMT and survival pathway [76]. Thus, the benefits of plant-produced anti-PD-L1, especially on sensitizing anoikis presented in this study would strengthen the possibility of using plants as an alternative production host to meet the therapeutic requirements globally at economical costs. Nevertheless, the anoikis sensitizing effect of plant-produced anti-PD-L1 should be further evaluated in an *in vivo* study.

## Conclusion

In conclusion, *N*. *benthamiana* plant cell-based platform feasibility was illustrated for the rapid expression and congregation of anti-PD-L1 IgG1 Atezolizumab. The present study showed similarities in PD-L1 binding efficacy and *in vitro* functional capabilities in comparison with mammalian cell-based antibody Tecentriq^®^, thereby inferring the potentiality of plant-produced Atezolizumab for use in cancer therapeutics. Further the plant produced Atezolizumab was assessed in *in vitro* studies on metastatic lung cancer cells with emphasis on anoikis sensitization and underlying regulatory mechanisms. Future studies in human knock-in mice models are essential to study the *in vivo* efficacy of plant-produced anti-PD-L1 Atezolizumab. Altogether, this study proves the reproducibility, scalability and cost-effectiveness of plant expression system as an alternative platform for large scale manufacturing approaches, producing mAb-based check point inhibitors for immunotherapy.

## Supporting information

S1 Fig Anoikis sensitizing activity in human non-small cell lung cancer H460 cells was further experimented with wild type crude extract of *N*. *benthamiana* (1.0 μg/mL) in comparison with Tecentriq®, a commercial Atezolizumab (1.0 μg/mL), and plant produced anti-PD-L1 (1.0 μg/mL). (A) Reduction in % cell viability analyzed by XTT assay (B) Flow cytometry plots of annexin V-FITC/propidium iodide (PI) showing anoikis induction in H460 cells. Augmented early apoptosis (Annexin V-FITC positive/PI negative) and late apoptosis (Annexin V-FITC positive/PI positive) was observed when cells were treated with plant anti-PD-L1 antibody and commercial Atezolizumab for 24 h under the detached condition. (C) The percentage of early and late apoptosis in H460 cells is depicted comparison with a commercial Atezolizumab, plant produced anti-PD-L1 and wild type plant extract. Values are means of independent triplicate experiments ± SD; *p < 0.05 compared with non-treated control cells.
(TIF)

S1 Raw images (PDF)
