# Supplementary figures and images for: Expression of plant-produced anti-PD-L1 antibody with anoikis sensitizing activity in human lung cancer cells via., suppression on epithelial-mesenchymal transition
Source: PLoS One. 2022 Nov 11;17(11):e0274737. doi: 10.1371/journal.pone.0274737 (PMC9651560; doi:10.1371/journal.pone.0274737)

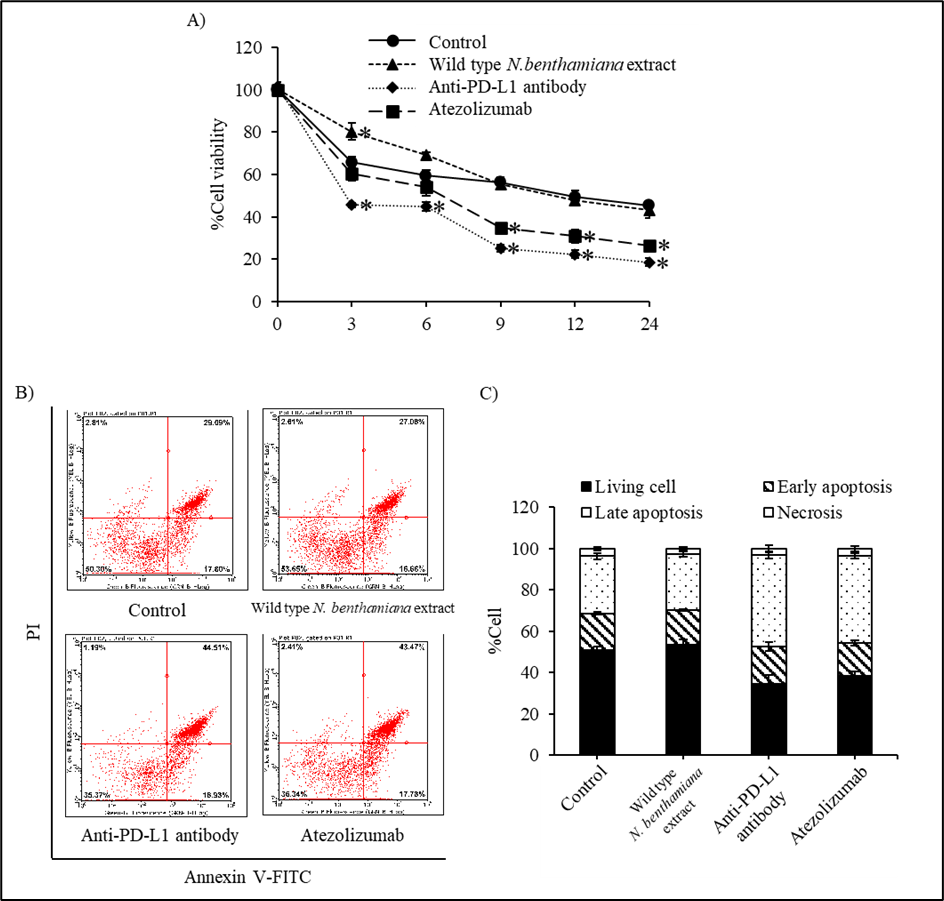

Supplement: S1 Fig — (A) Reduction in % cell viability analyzed by XTT assay (B) Flow cytometry plots of annexin V-FITC/propidium iodide (PI) showing anoikis induction in H460 cells. Augmented early apoptosis (Annexin V-FITC positive/PI negative) and late apoptosis (Annexin V-FITC positive/PI positive) was observed when cells were treated with plant anti-PD-L1 antibody and commercial Atezolizumab for 24 h under the detached condition. (C) The percentage of early and late apoptosis in H460 cells is depicted comparison with a commercial Atezolizumab, plant produced anti-PD-L1 and wild type plant extract. Values are means of independent triplicate experiments ± SD; *p < 0.05 compared with non-treated control cells. (TIF) [file pone.0274737.s001.tif]
