# Supplementary material for: Expression of plant-produced anti-PD-L1 antibody with anoikis sensitizing activity in human lung cancer cells via., suppression on epithelial-mesenchymal transition
Source: PLoS One. 2022 Nov 11;17(11):e0274737. doi: 10.1371/journal.pone.0274737 (PMC9651560; doi:10.1371/journal.pone.0274737)

Supplementary Raw Data

Figure S1. SDS PAGE & Western Blot of Plant-produced Atezolizumab

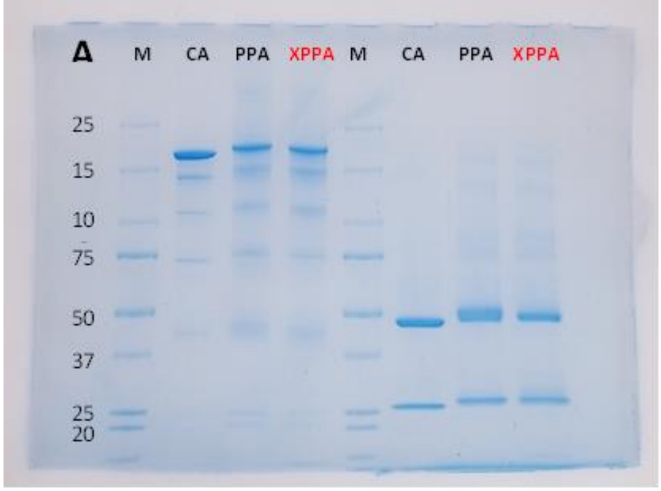

A: SDS gel picture stained with Coomassie

B: Western blot analysis with sheep anti-human gamma-HRP under Non-reducing and reducing conditions

C: Western blot analysis with sheep anti-human kappa-HRP under Non-reducing and reducing conditions

M: Protein ladder

CA: Commercial Atezolizumab

PPA: Plant-produced Atezolizumab

XPPA: Plant-produced Atezolizumab loaded at different concentration but not included in the Manuscript file

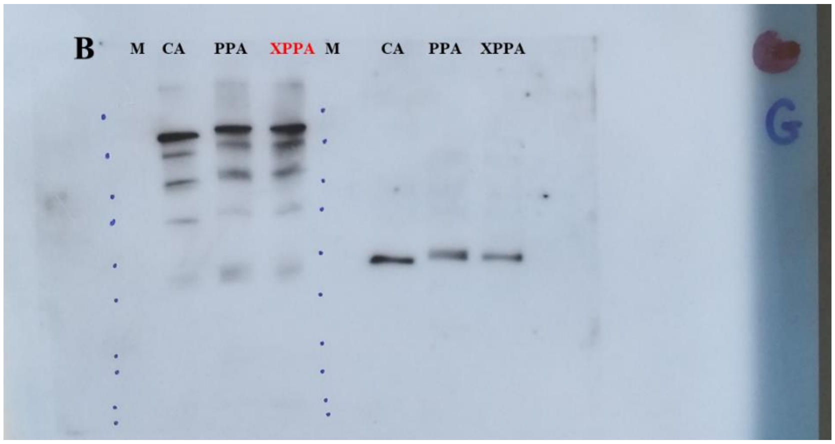

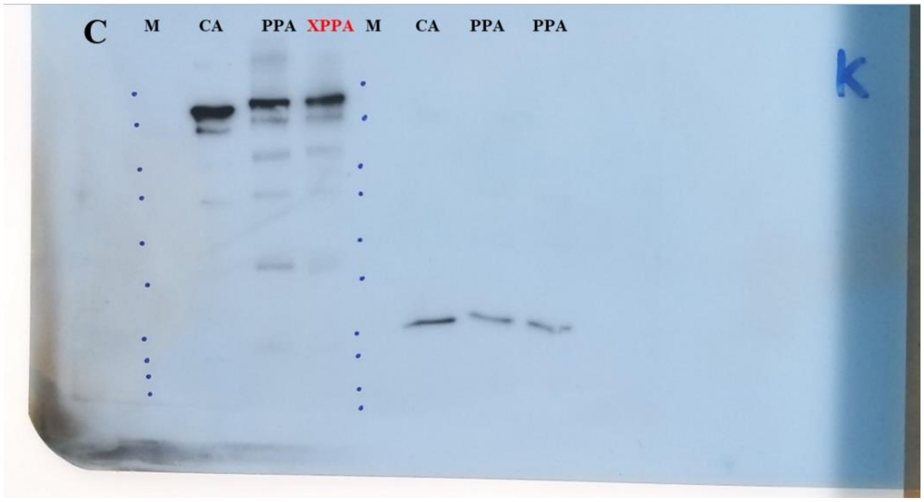

Supplement: S1 Raw images — (PDF) [file pone.0274737.s002.pdf]
